# Caloric Restriction Preserves BBB Integrity After Transient Focal Cerebral Ischemia Through Reducing Neutrophil Infiltration

**DOI:** 10.1111/cns.70257

**Published:** 2025-02-06

**Authors:** Chenran Wang, Leilei Mao, Miao He, Jia Zhang, Yichen Huang, Yue Zhang, Jing Xu, Shaoqiang Huang, Yanqin Gao

**Affiliations:** ^1^ Department of Anesthesiology of Eye & Ent Hospital, Department of Anesthesiology of Obstetrics & Gynecology Hospital, State Key Laboratory of Medical Neurobiology, MOE Frontiers Center for Brain Science, and Institutes of Brain Science Fudan University Shanghai China

**Keywords:** blood–brain barrier, caloric restriction, neutrophils, stroke

## Abstract

**Aims:**

Caloric restriction is a health‐promoting lifestyle that has been reported to protect both white and gray matter in cases of ischemic stroke. This study will explore the underlying mechanism of restricted feeding (RF) and provide a theoretical basis for precise clinical treatment of stroke.

**Methods:**

In this study, we pretreated C57BL/6J mice with 70% RF for a continuous 28‐day period prior to 60 min of transient focal cerebral ischemia (tFCI). Histological staining, diffusion tensor imaging (DTI), and behavioral assessments were used to assess RF's neuroprotection following tFCI. Immunofluorescence staining, quantitative real‐time PCR, and flow cytometry were conducted to evaluate brain inflammation post‐tFCI. Western blot, immunofluorescence staining, tracers, and electric microscopy were used to assess the blood–brain barrier (BBB) integrity. Peripheral neutrophils were cleared by administrating an anti‐Ly6G antibody.

**Results:**

Initially, DTI, NeuN staining, and a batch of behavioral tests verified that RF significantly mitigated both gray/white matter injury and neurological deficits in the short‐ and long‐term following tFCI. RF mice showed more anti‐inflammatory microglia in their brains, along with reduced inflammatory cytokines, and chemokines. Interestingly, RF significantly reduced the neutrophils and macrophage infiltration. Subsequently, we observed that RF mice exhibited better BBB integrity following tFCI, with reduced neutrophil infiltration and matrix metalloprotein‐9 release. Furthermore, the clearance of neutrophils with anti‐Ly6G antibody in ad libitum feeding mice (LF‐Ly6G) elicited comparable neuroprotective effects to those observed in RF, including improvements in neurological deficits, reductions in infarct volume, and mitigation of BBB damage.

**Conclusions:**

In summary, our findings suggest that RF maintains the BBB integrity following ischemic stroke at least partially by reducing neutrophil infiltration, thereby alleviating both neurological and histological impairments.

## Graphical abstract

RF maintains the BBB integrity following ischemic stroke by reducing neutrophil infiltration and the subsequent release of MMP9, thereby alleviating both neurological and histological impairments.

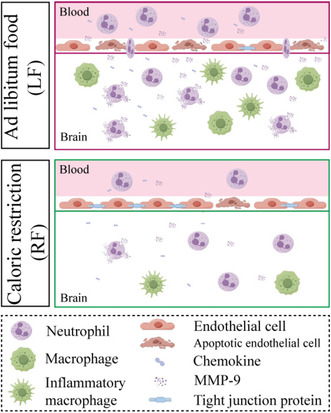

## 1 Introduction

Stroke is the second most common cause of death and the third most common cause of disability around the world, with 1 in 4 people expected to experience a stroke during their lifetime [1]. Ischemic stroke constitutes 60%–70% of all strokes and results from arterial occlusion [2, 3]. Ischemic stroke represents a significant contributor to adult disability, with approximately 50% of survivors requiring assistance with at least one activity of daily living. Beyond the physical dependence and disability associated with stroke, the resultant cumulative brain damage from recurrent incidents leads to cognitive decline. This condition imposes considerable burdens on families and society, particularly as the population continues to age [3, 4, 5]. At present, the temporal constraints and associated complications of thrombolysis and thrombectomy significantly restrict their applicability [6, 7, 8]. Moreover, the majority of neuroprotective agents designed for the treatment of ischemic stroke have not succeeded in clinical trials [9, 10]. Consequently, it is imperative to develop efficacious preventive strategies aimed at minimizing the incidence of ischemic stroke or mitigating cerebral damage following stroke.

Caloric restriction involves reducing calorie intake without inducing malnutrition. It is a health‐promoting lifestyle that has been shown to protect against various diseases, including diabetes, obesity, neurodegenerative disorders, as well as cerebral vascular diseases [11, 12, 13], delay aging, and extend lifespan in organisms such as *Caenorhabditis elegans* , *Drosophila*, rodents, and non‐human primates [14, 15]. The underlying mechanism of restricted feeding (RF)'s protective effects includes downregulating insulin/IGF1, mTORC1, activating GCN2 and sirtuins, resisting oxidative stress, and changing gut microbial composition [11, 16, 17]. Previous research indicates that preconditioning with caloric restriction provides long‐term protection against both gray and white matter injury following transient focal cerebral ischemia (tFCI) [18, 19]. However, the underlying mechanisms remain to be fully investigated.

Caloric restriction has been reported to improve cerebrovascular health by increasing the resistance to oxidative stress, enhancing nitric oxide bioactivity, and suppressing vascular inflammation [20]. In an aging mouse model, caloric restriction preserves cerebral blood flow (CBF) at 20 months of age compared to normally fed counterparts [21]. In another model involving a high‐fat diet, caloric restriction pretreatment attenuated the blood–brain barrier (BBB) leakage [22]. Caloric restriction enhances tight junction protein expression in brain endothelial cells by altering intestinal flora short‐chain fatty acids, thus maintaining BBB integrity and function [23, 24]. Early after ischemic stroke, due to the increased BBB permeability, immune cells and molecules in circulation infiltrated into the brain parenchyma, aggravating inflammation and brain edema [25, 26, 27]. Given that BBB dysfunction is a critical pathophysiological mechanism following ischemic stroke, we hypothesize that caloric restriction protects the neurological function in ischemic stroke by preserving BBB integrity.

In this study, we pretreated 8‐week‐old C57BL/6J male mice with 70% of ad libitum food regimen as RF for 4 weeks prior to inducing tFCI. First, we verified the protective effect of caloric restriction on ischemic stroke. Then, we observed that RF led to a downregulation of inflammatory factors and chemokines within the brain postischemic stroke, accompanied by a reduction in peripheral immune cell infiltration, such as neutrophils and macrophages. Subsequently, we demonstrated that caloric restriction enhanced the preservation of BBB integrity following ischemic stroke. The application of an anti‐Ly6G antibody to inhibit neutrophil infiltration could mimic the protective effects of RF on ischemic stroke. Our study demonstrates that caloric restriction preserves BBB integrity following tFCI by decreasing neutrophil infiltration.

## 2 Methods

### 2.1 Animal Models of tFCI and RF

Male C57BL/6J mice aged 8–10 weeks were sourced from SiPeiFu Animal Center in Beijing, China. Animals were housed in facilities with regulated temperature and humidity, following a 12‐h light–dark cycle. Animal experiments were approved by Fudan University's Animal Care and Use Committee and conducted in compliance with the ARRIVE guidelines (Approval Numbers: 20120119–120, 202409035S). Efforts were made to minimize animal suffering and usage. Previously described protocols were followed for dietary regimens [18]. Briefly, mice on a limited diet were given 70% of the usual diet (2.38 g ± 0.14 g per mouse, RF) each day for 4 weeks prior to tFCI surgery, whereas mice fed freely received an adequate rodent diet (3.4 g ± 0.14 g per mouse, LF) [18]. During the RF regimen, food was administered every 24 h to ensure the provision of 30% reduction in food intake every day.

The tFCI mice model was established using middle cerebral artery occlusion, as previously detailed [18]. In brief, mice were anesthetized using 1%–2% isoflurane in a 70% N_2_/30% O_2_ mixture, followed by preparation for insertion, which involved isolating the common carotid, internal carotid, and external carotid arteries. A 7–0 nylon monofilament with a silicone‐coated tip (0.19–0.21 mm in diameter) was inserted into the external carotid artery and moved 1.8 cm up the internal carotid artery to occlude the middle cerebral artery. After 60 min of ischemia, the filament was removed to allow reperfusion, excluding animals with less than a 75% reduction in CBF as determined by laser Doppler flowmetry. Sham‐operated mice were anesthetized and underwent surgery without tFCI. Behavioral tests were assessed by investigators who were blind to the group assignments.

### 2.2 Neurobehavioral Tests

This study assessed sensory motor dysfunctions in mice using the neurological severity score, grid‐walking test, and wire‐hanging test on 1, 3, 5, 7, 14, 21, 28, and 35 days post‐tFCI. Neurological severity score was conducted using modified Garcia score, which is based on a number of individual tests, including body proprioception, spontaneous activity, limb symmetry, lateral turning, forelimb walking, and climbing. Scores were derived by totaling the results of six subtests, each scored between 0 and 3. In the grid‐walking test, each mouse was positioned on a stainless‐steel grid floor (20 cm × 40 cm with a mesh size of 4 cm^2^). The number of forepaw foot fault was recorded during 3‐min period. In the wire‐hanging test, mice were positioned at the center of a wire (50 cm in length, 2 mm in width, and 40 cm in height) and observed for 1 min, with a 5‐min interval between each of the three trials. The scoring for a mouse was as follows: 0 for falling off; 1 for hanging with both forepaws; 2 for hanging with climbing attempts; 3 for hanging with both forepaws and one or two hind paws; 4 for hanging with all paws and tail wrapped; and 5 for escaping to a platform.

Long‐term cognitive deficits were assessed using the Morris water maze test, as previously described [28]. In the learning phase, four daily trials were carried out between Days 28 and 33. The duration it took for the mouse to reach the platform during the learning test was recorded to evaluate spatial learning skills. In the memory test, a 60‐s probe trial took place without the platform, and the distance in the goal quadrant was measured to assess spatial memory.

### 2.3 Immunohistochemistry

Following execution, mice were perfused with ice‐cold saline followed by 4% paraformaldehyde. Brains were extracted and sequentially immersed in 4% paraformaldehyde, 20% sucrose, and 30% sucrose for postfixation and dehydration, then sectioned into 25 μm coronal slices using a cryostat. Brain slices were washed with PBS, blocked with 5% donkey serum, and incubated in primary antibody at 4°C overnight. After washing, slices were incubated in secondary antibody for 1 h, followed by mounted with DAPI Fluoromount‐G. The brain slice images were captured using a Nikon A1 confocal microscope and analyzed with ImageJ software. The primary antibodies utilized were antiglial fibrillary acidic protein (anti‐GFAP, ab4674, Abcam), anti‐Iba1 (ab5076, Abcam), anti‐MMP‐9 (ab38898, Abcam), anti‐Ly6G (MAB1037, R&D), anti‐Arg1 (sc271430, Santa Cruz), anti‐CD31 (AF3628, R&D), anti‐ZO‐1 (ab216880, Abcam), anti‐CD16/32 (ab25235, Abcam), and anti‐NeuN (ab190195, Abcam). All the fluorescence secondary antibodies were purchased from Jackson ImmunoResearch (USA).

### 2.4 Measurement of Tissue Loss

2,3,5‐Triphenyltetrazolium chloride (TTC) staining was performed 2 days after tFCI. Brain slices, each 1 mm in thickness, were immersed in prewarmed 2% TTC solution in saline for a duration of 10 min. Subsequently, we selected 10 brain slices from each brain at various levels (bregma 0.5 mm to −1.9 mm) for MAP2 staining to assess brain atrophy. The area of brain atrophy in each slice was determined by subtracting the ipsilateral noninfarcted area from the contralateral brain area. The total atrophy volume was the sum of these areas across all slices. The atrophied area or volume proportion was calculated relative to the contralateral side.

An in vivo MRI scan was conducted with an 11.7 T small‐animal MRI system (Bruker Biospin, Billerica, MA, USA) at 3 and 14 days post‐tFCI. A diffusion tensor imaging (DTI) scan was used to assess the microstructure of cerebral white matter. Following perfusion with saline and paraformaldehyde, mice brains were fixed in paraformaldehyde fix solution for 24 h. Subsequently, the brains were air‐dried, placed in test tubes with carbon‐free oil, and scanned using the machine's coils. The primary parameters were set as follows: FOV = 20 × 20 mm, TR/TE = 4000/17.33 ms, and slice thickness = 0.4 mm.

### 2.5 Detection of BBB Disruption

Following tFCI, Evans blue (EB) extravasation, fluorescence tracer leakage, and endogenous plasma IgG leakage were used to assess BBB permeability [28]. Mice received an intravenous injection of 4% EB 72 h post‐tFCI while under anesthesia. Two hours post‐EB exposure, mice were reanesthetized for intracardiac perfusion with PBS, and the ipsilateral brain tissue was collected. The ipsilateral hemisphere was then weighed, homogenized, and incubated at 4°C for 24 h in 50% trichloroacetic acid (TCA). Postcentrifugation, the absorption of the supernatant was measured at 620 nm with spectrophotometer (Bio‐Rad). The EB content was valued as μg/g of brain tissue by using a standard curve.

For assessing cadaverine and IgG leakage, mice received an injection of 200 μL Alexa Fluor 555 cadaverin (0.95 kDa, Invitrogen, 1 μg/μL) into the femoral vein 1 h prior to euthanasia. Twenty‐five micrometer coronal slices were prepared by a frozen sectioning machine, and these sections were either directly used for fluorescence detection or labeled with immunofluorescence for IgG. Refer to the Immunofluorescence Staining section for detailed methodologies pertaining to image acquisition and analysis.

### 2.6 Flow Cytometry

Three days after tFCI or sham, immune cell populations in the brain, peripheral blood, and spleen were analyzed using flow cytometry. Following established protocols [29], blood was collected with an anticoagulant, after which mice were perfused with chilled Hank's balanced salt solution (HBSS), and their spleens and the ipsilateral brains were promptly harvested. Brain tissue was minced and enzymatically processed into a single cell suspension using the Neural Tissue Dissociation Kit (P) (130–092‐628, Miltenyi Biotec). The brain‐infiltrating leukocytes were enriched by density gradient centrifugation (30%/70% percoll gradient). The spleens were crushed and passed through a 70 μm cell strainer. Red blood cells in the spleens or blood were broken down using ACK buffer. Cell pellets were collected postcentrifugation at 500 g for 5 min at 18°C.

Cell suspensions in the final 100 μL of HBSS +2% FBS were washed with HBSS, blocked with rat anti‐CD16/32 antibody (553,142, BD Biosciences) for 10 min, followed by staining with fluorophore‐conjugated antibodies (4°C for 30 min). These antibodies included: CD45‐efluor450 (48–0451‐82, Thermo Fisher Scientific), CD11b‐APC‐Cy7 (47–0112‐82, Thermo Fisher Scientific), CD3‐APC (17–0032‐82, Thermo Fisher Scientific), Gr1(Ly6G)‐PE (12–9668‐82, Thermo Fisher Scientific), CD11c‐PerCP‐Cy5.5 (45–0114‐82, Thermo Fisher Scientific), and CD19‐FITC (11–0193‐82, Thermo Fisher Scientific). Flow cytometry was conducted using the Beckman Coulter CytoFLEX, and FlowJo v10 software was utilized for data analysis.

### 2.7 Quantitative Real‐Time PCR

Total RNA was extracted from the ipsilateral brain tissue utilizing Trizol reagent (19201ES60, Yisen). Following this, cDNA was synthesized from 1 μg of mRNA using the First Strand cDNA Synthesis Kit (K1622, Thermo Fisher Scientific), according to the manufacturer's instructions. The quantitative real‐time PCR was performed on a 7500 Real‐Time PCR System using HifTM QPCR SYBR Green Master Mix (11201ES08, Yeasen) as the detection dye. The PCR protocol began with an initial denaturation at 95°C for 5 min, succeeded by 40 cycles (95°C for 10 s, 55°C for 20 s, and 72°C for 20 s). The relative expression levels of the candidate genes were evaluated using the 2^–ΔΔCt^ method. The primer sequences used in this study were as follows:

**Table:** 

Gene		Sequence (5′‐3′)
*Il1b*	Forward	CTCCATGAGCTTTGTACAAGG
	Reverse	TGCTGATGTACCAGTTGGGG
*Nos2*(iNOS)	Forward	CAAGCACCTTGGAAGAGGAG
	Reverse	AAGGCCAAACACAGCATACC
*Gapdh*	Forward	AGGTCGGTGTGAACGGATTTG
	Reverse	TGTAGACCATGTAGTTGAGGTCA
*Tnfα*	Forward	GACCCTCACACTCAGATCATCTTCT
	Reverse	CCTCCACTTGGTGGTTTGCT
*Cx3cl1*	Forward	ACCTATGGCCCTGACATCATCAC
	Reverse	CTTGCCAGCCCTCAGAATCAC
*Cxcl12*	Forward	GCTCCCTTGGTTCAGAAAATTG
	Reverse	TCACCAGACAGGTGCCATCA
*Ccl2*	Forward	CACTCACCTGCTGCTACTCA
	Reverse	GCTTGGTGACAAAAACTACAGC
*Ccl3*	Forward	CATGACACTCTGCAACCAAGTCTTC
	Reverse	GAGCAAAGGCTGCTGGTTTCA
*Ccl7*	Forward	GCTGCTTTCAGCATCCAAGTG
	Reverse	CCAGGGACACCGACTACTG
*Ccl22*	Forward	CTGATGCAGGTCCCTATGGT
	Reverse	GCAGGATTTTGAGGTCCAGA
*Il6*	Forward	ACACATGTTCTCTGGGAAATC
	Reverse	AGTGCATCATCGTTGTTCATA

### 2.8 Western Blot

Proteins were extracted from the ipsilateral brain tissue using cold RIPA buffer, and 30 ug of protein was loaded onto SDS‐PAGE gels for transfer to PVDF membranes. After a 1.5‐h blocking period with 5% BSA, the PVDF membrane was incubated overnight at 4°C with the following primary antibodies: anti‐MMP9 (ab38898, Abcam), anti‐ZO‐1 (ab216880, Abcam), anti‐cadherin 10 (ab134137, Abcam), and anti‐β‐actin (30101ES50, Yeasen). The following day, samples were incubated for 1 h at room temperature with HRP‐conjugated secondary antibodies from Cell Signaling Technology. An ECL chemiluminescence development system was used to create the images, which were then captured with a Bio‐Rad ChemiDoc developer (Bio‐Rad, USA). The expression of target proteins was quantified using ImageJ software and normalized against β‐actin for relative expression.

### 2.9 Transmission Electron Microscopy

Brain tissue (thickness < 1 mm) was fixed in 2.5% glutaraldehyde at 4°C for 24 h, dehydrated with graded ethanol and acetone, embedded in epoxy resin, and sectioned into 50 μm slices. These ultrathin sections were then stained with 3% uranium acetate and lead citrate. Subsequently, the images were captured by Philips CM120 electron microscope and analyzed.

### 2.10 Neutrophils Depletion

To mitigate peripheral neutrophil infiltration into the brain, we administered Ly6G antibody (100 μg per dose) or an equivalent amount of IgG as an isotype control to mice via intraperitoneal injection. This treatment was conducted 1 day prior to surgery and subsequently on Days 1, 3, 5, and 7 postsurgery. The anti‐Ly6G antibody effectively blocked neutrophil infiltration as our previous study shows [30].

### 2.11 Statistical Analysis

Data analysis was conducted using SPSS Statistics 20 and GraphPad Prism 10 with results expressed as mean ± standard error of measurement (Mean ± SEM). The Shapiro–Wilk test was employed to assess the normality of all data. For data that adhered to a normal distribution, an unpaired two‐tailed Student's *t*‐test was used to compare two groups. One‐way or two‐way ANOVA, accompanied by Bonferroni post hoc testing, was utilized to assess differences among three or more groups. For nonnormally distributed data, Mann–Whitney U test was employed to compare between two groups, and the Kruskal–Wallis test with Dunn's multiple‐comparison post hoc test was applied for data involving more than two groups. *p*‐Values < 0.05 were considered statistically significant.

## 3 Results

### 3.1 RF Alleviates Short‐Term and Long‐Term Histological and Neurological Deficits After Ischemic Stroke

We pretreated 8‐week‐old C57BL/6J mice with either 70% caloric restriction (RF group) or ad libitum feeding (LF group) for 28 consecutive days. After 4 weeks, mice subjected to 70% caloric restriction (RF group) exhibited lower body weights than those in the LF group before surgery (Figure S1A), demonstrating effective weight reduction. Then, tFCI model was conducted for 60 min followed by reperfusion. The relative CBF was not different between LF and RF mice (Figure S1B&S1C, the blood flow in ipsilateral/contralateral, Laser Speckle Contrast Imaging System, Perimed Inc). The mortality rate observed in the LF group was determined to be 25% throughout the duration of our study. In contrast, no fatalities were observed in the RF group, attributable to the protective effects of the restricted diet (Figure S1D).

**FIGURE 1:**
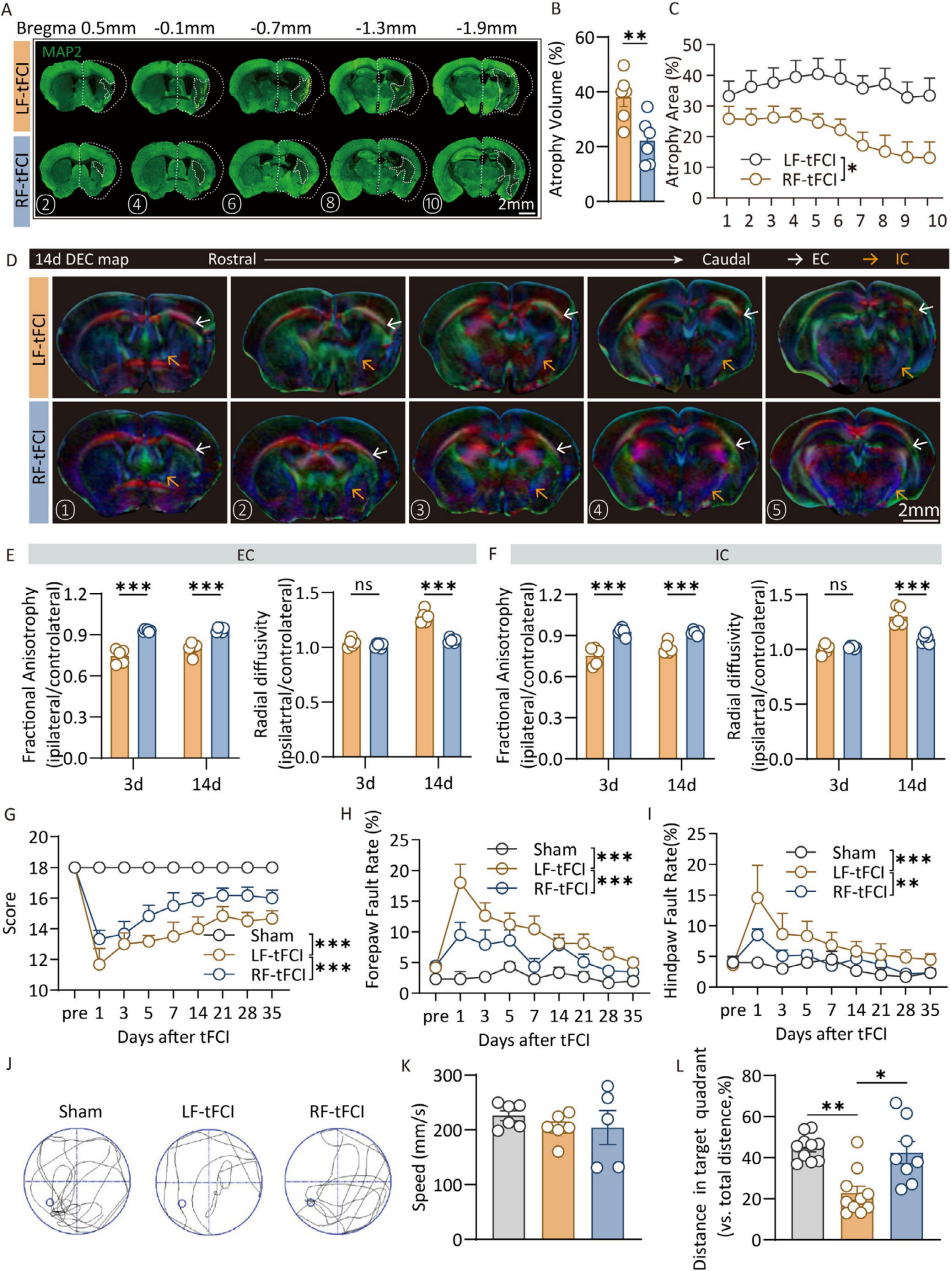
Caloric restriction ameliorates histological and neurological deficits following ischemic stroke. (A) Representative images of MAP2 immunofluorescence staining. (B, C) The total atrophy volume (B) and the atrophy area (C) of each layer from rostral to caudal at 28 days after tFCI. *n* = 6/group. (D) DEC map presented was used to visualize in vivo DTI on Day 14 after tFCI. Scale bar: 3 mm. (E, F) Statistical plots illustrate the variations in FA and RD within the EC (E) and IC (F) regions on Days 3 and 14 post‐tFCI. *n* = 5/group. (G–I) Sensorimotor deficits were evaluated before (pre) and up to 35 days after tFCI or Sham surgery by Garcia score (G) and grid‐walking test (H, I). *n* = 6/group. (J–L) Morris water maze test showed the tracking plot of (J), swimming speed (K), and the percentage of distance traveled in target quadrant (L) in test. *n* = 8–10/group. All data are presented as means ± SEM. Data were analyzed using unpaired two‐tail Student's *t*‐test (B, C), one‐way (K, L) or two‐way (E, F, G–I) ANOVA followed by Bonferroni post hoc test, and Kruskal–Wallis test with Dunn's multiple‐comparison post hoc test (L). *p < 0.05, **p < 0.01, ***p < 0.001, ns, no significance, as indicated.

To further determine the protective effect of RF on tissue damage in the acute phases after ischemic stroke, TTC staining was performed to distinguish the infarct core and viable tissue 2 days after tFCI (Figure S3A). It showed that RF preconditioning significantly reduced the infarct area and volume in the brain following ischemic stroke (Figure S3B,C). Fluoro‐Jade C staining was used to assess neuronal degeneration. Significant neuronal degeneration was detected in the cortex and striatum 48 h post‐tFCI. Notably, the RF treatment group showed a marked decrease in degenerating neurons relative to the LF group (Figure S3D,E). Long‐term neuronal death following ischemic stroke resulted in brain atrophy, as shown by MAP2 immunostaining 28 days after tFCI (Figure 1A). The RF group exhibited a significantly lower percentage of atrophy volume compared to the LF group (Figure 1B,C). To assess the integrity of white matter after tFCI, DTI was employed on Days 3 and 14. Directionally encoded color (DEC) maps revealed that the white matter fibers in the internal capsule (IC) and external capsule (EC) in RF mice demonstrated a higher fiber density compared to LF mice (Figure 1D). Elevated fractional anisotropy (FA, indicating white matter structure) was observed in RF mice on Days 3 and 14 post‐tFCI, along with a notable decrease in radial diffusivity (RD, indicating demyelination or axonal swelling) on Day 14 post‐tFCI (Figure 1E,F; Figure S2). GFAP staining was performed 28 days after tFCI to evaluate the severity of glial scarring. Ischemic stroke led to long‐term glial scarring, which was significantly reduced by RF (Figure S3F,G). These findings suggested that caloric restriction preconditioning effectively mitigates both short‐term and long‐term histological deficits after ischemic stroke.

Sensorimotor and cognitive dysfunctions are among the most prevalent complications of ischemic stroke. Therefore, we conducted several behavioral tests to evaluate the sensorimotor and cognitive function following tFCI. The Garcia Neurological Scoring Scale was employed to assess neurological deficits, revealing that RF mice achieved higher scores than LF mice (Figure 1G). Sensorimotor deficits were evaluated using the grid‐walking test, where RF mice exhibited a reduced fault rate in both the forepaw and hind paw compared to LF mice post‐tFCI (Figure 1H,I). The Morris water maze was used to evaluate memory ability in chronic phase during 28–34 days post‐tFCI. Although no significant difference in swim speed was observed among Sham, LF, and RF mice (Figure 1J,K), LF mice covered a shorter distance in the target quadrant (as a percentage of total distance) during the test day compared to both Sham and RF mice, which indicated that the memory of RF mice was better than LF mice (Figure 1L).

Taking together, we confirmed that RF alleviated short‐term and long‐term histological and neurological deficits after ischemic stroke, consistent with previous research [18].

### 3.2 RF Reduces Inflammatory Cytokines and Chemokines in the Brain After Ischemic Stroke

**FIGURE 2:**
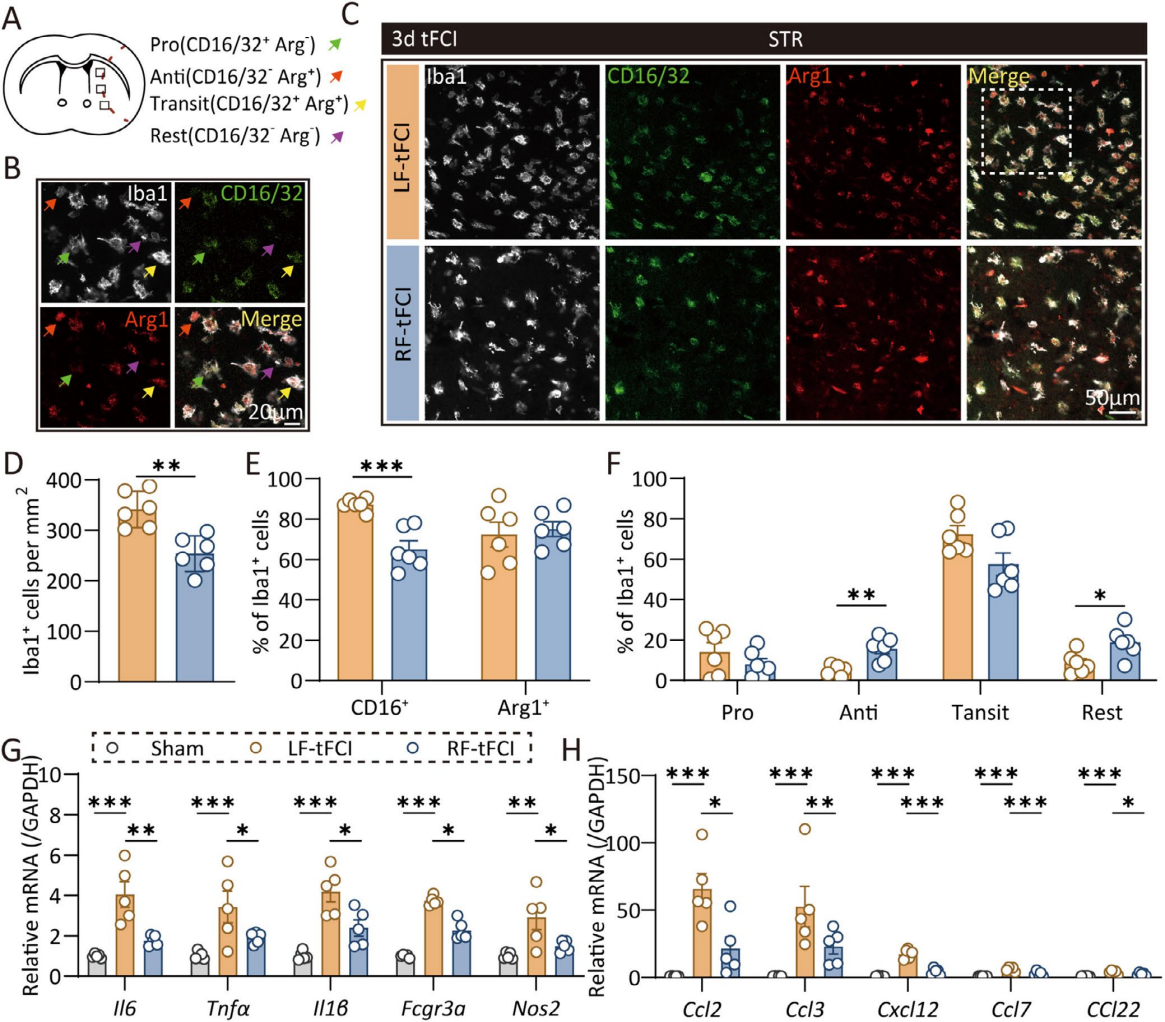
Caloric restriction modulates microglia heterogeneity, and the expression of inflammatory cytokines, and chemokines in short term after ischemic stroke. (A) Schematic diagram indicating the regions where images were captured in (C). (B) Representative images of triple immunostaining (Iba1, CD16/32, and Arg1) taken from the dashed box in Figure 2C. The green arrow indicated promicroglia, red arrow indicated antimicroglia, yellow arrow indicated transit microglia, and purple arrow indicated rest microglia. Scale bar: 20 um. (C) Representative images of Iba1, CD16/32, and Arg1 immunofluorescence staining in striatum 3 days after tFCI. Scale bar: 50 um. (D) The density of Iba1^+^ cells. *n* = 6/group. (E‐F) The percentage of different microglia phenotypes accounted for Iba1^+^ cells. *n* = 6/group. (G‐H) The relative expression of proinflammatory cytokines (G) and chemokines (H). *n* = 5/group. All data are presented as means ± SEM. Data were analyzed using unpaired two‐tail Student's *t*‐test (D–F), one‐way ANOVA followed by Bonferroni post hoc test (H), and Kruskal–Wallis test with Dunn's multiple‐comparison post hoc test (G). *p < 0.05, **p < 0.01, and ***p < 0.001, as indicated.

Inflammation is a critical pathophysiological response following ischemic stroke. Microglia, as the resident immune cells in the brain parenchyma, are the initial responders following ischemia/reperfusion. Upon activation, they express various inflammatory cytokines and chemokines, which facilitate the phagocytosis of dead cells and debris, promote tissue restoration, and recruit peripheral immune cells to the brain [31]. To investigate microglial activation, we performed costaining of CD16/32, a proinflammatory marker; Arg1, an anti‐inflammatory marker; and Iba1, a microglia/macrophage marker 3 days post‐tFCI. According to previous research, microglia exhibit significant heterogeneity and can be categorized into four phenotypes briefly: proinflammatory (Pro, Iba1^+^/CD16/32^+^Arg1^−^), anti‐inflammatory (Anti, Iba1^+^/CD16/32^−^Arg1^+^), transitional (Transit, Iba1^+^/CD16/32^+^/Arg1^+^), and rest (Rest, Iba1^+^/CD16/32^−^/Arg1^−^), as shown in Figure 2A–C. The density of Iba1^+^ cells was lower in RF mice than in LF mice (Figure 2D), with a decreased proportion of Iba1^+^/CD16/32^+^ cells among the total Iba1^+^ cells in RF mice (Figure 2E). RF mice demonstrated a greater proportion of Anti and Rest microglial phenotypes than LF mice, whereas the Pro and Transit phenotypes were less prevalent in RF mice compared to LF mice (Figure 2F). These findings indicated that RF modulated microglial heterogeneity following ischemic stroke.

To further evaluate brain inflammation post‐tFCI, we used qPCR to detect inflammatory cytokines in peri‐infarct tissue 3 days after tFCI. The concentrations of *Il‐6, Tnfα, Il‐16, Fcgr3α*, and *Nos2* were elevated following ischemia/reperfusion, which were partially mitigated by RF (Figure 2G). In addition to inflammatory cytokines, microglia also express numerous chemokines to recruit peripheral immune cells during disease. Therefore, we also assessed the expression of chemokines in peri‐infarct tissue by qPCR. We observed upregulated gene expression of *Ccl2, Ccl3, Ccl7*, and *Ccl22* at 3 days postischemia/reperfusion. Similarly, RF mice exhibited lower expression levels of these chemokines compared to LF mice (Figure 2H).

Collectively, these data suggested that RF modulated microglial heterogeneity and reduced the expression of several inflammatory cytokines and chemokines during the acute phase following ischemic stroke.

### 3.3 RF Reduces Immune Cells Infiltration Into the Brain After Ischemic Stroke

**FIGURE 3:**
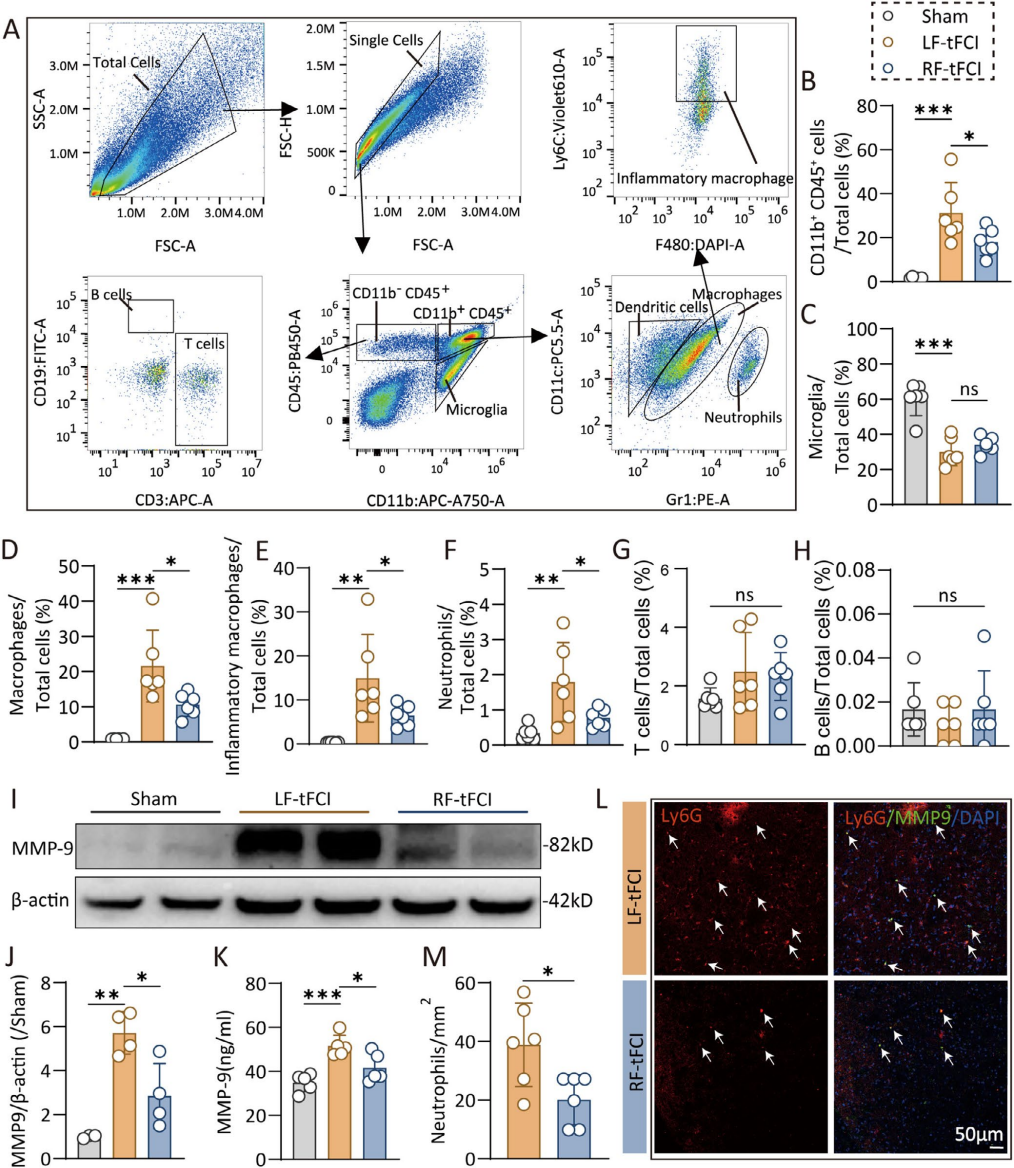
Caloric restriction preconditioning reduces peripheral neutrophils and macrophages infiltration in acute phase after ischemic stroke. (A) Gating strategy of flow cytometry that analyzes the infiltrated immune cells in brain 3 days after tFCI. (B–H) The percentage of CD11b^+^CD45^+^ cells (B), microglia (C), macrophages (D), inflammatory macrophages (E), neutrophils (F), T cells (G), and B cells (H) accounted for total cells. *n* = 6/group. (I) Western blot of the penumbra and core tissue 3 days after tFCI were probed with anti‐MMP9 and β‐actin. (J) Quantification of relative MMP9 protein expression in western blot. *n* = 4/group. (K) ELISA analysis of MMP9 in brain 3 days after tFCI. *n* = 5/group. (L‐M) Representative images (L) and quantification (M) of Ly6G/MMP9/DAPI immunofluorescence staining 3 days after tFCI. Scale bar, 50 um. *n* = 6/group. All data are presented as means ± SEM. Data were analyzed using one‐way ANOVA followed by Bonferroni post hoc test (C, G, J, K), Kruskal–Wallis test with Dunn's multiple‐comparison post hoc test (B, D–F, H), and Mann–Whitney test (M). *p < 0.05, **p < 0.01, and ***p < 0.001; ns, no significance, as indicated.

Chemokines are cytokines that play a crucial role in recruiting immune cells to specific tissues for the purposes of phagocytosis, antigen clearance, and removal of debris or dead cells. In Figure 2H, we observed RF significantly reduced the gene expression of several chemokines after ischemic stroke. We performed flow cytometry 3 days post‐tFCI to analyze infiltrated immune cells in the brain, employing a gating strategy for neutrophils (CD11b^+^CD45^hi^CD11c^+^Gr1^+^), microglia (CD11b^+^CD45^int^), macrophages (CD11b^+^CD45^hi^), inflammatory macrophages (CD11b^+^CD45^hi^Ly6C^+^), T cells (CD11b^−^CD45^hi^CD3^+^), and B cells (CD11b^−^CD45^hi^CD19^+^) (Figure 3A). Although the microglia percentage was similar in RF and LF mice, RF mice had fewer macrophages, aligning with immunofluorescence staining results that indicated a reduced number of Iba1^+^ cells in RF mice (Figure 2D). Interestingly, RF mice exhibited decreased infiltration of immune cells, including neutrophils, macrophages, and inflammatory macrophages, into the brain compared to LF mice (Figure 3B–H).

Matrix metalloproteinase 9 (MMP9), a zinc metalloproteinase enzyme involved in extracellular matrix degradation and primarily expressed by neutrophils, was significantly upregulated following ischemic stroke [32]. However, RF attenuated the upregulation of MMP9 after tFCI (Figure 3I,J), a result corroborated by ELISA data (Figure 3K). Costaining of Ly6G, a neutrophil marker, revealed fewer neutrophils infiltrating the brain in RF mice compared to LF mice (Figure 3L,M), while there were no neutrophils infiltrating the normal brain [33](data are not shown). Additionally, there is no difference in the immune response of peripheral blood and spleen 3 days after tFCI between RF and LF mice analyzed by flow cytometry (Figure S4).

In summary, RF reduced neutrophil and macrophage infiltration into the brain without affecting immune responses in the blood and spleen following ischemic stroke. Consistent with the reduced neutrophil infiltration, MMP9 levels were also lower in RF mice compared to LF mice.

### 3.4 RF Preserves the Integrity of the BBB After Ischemic Stroke

**FIGURE 4:**
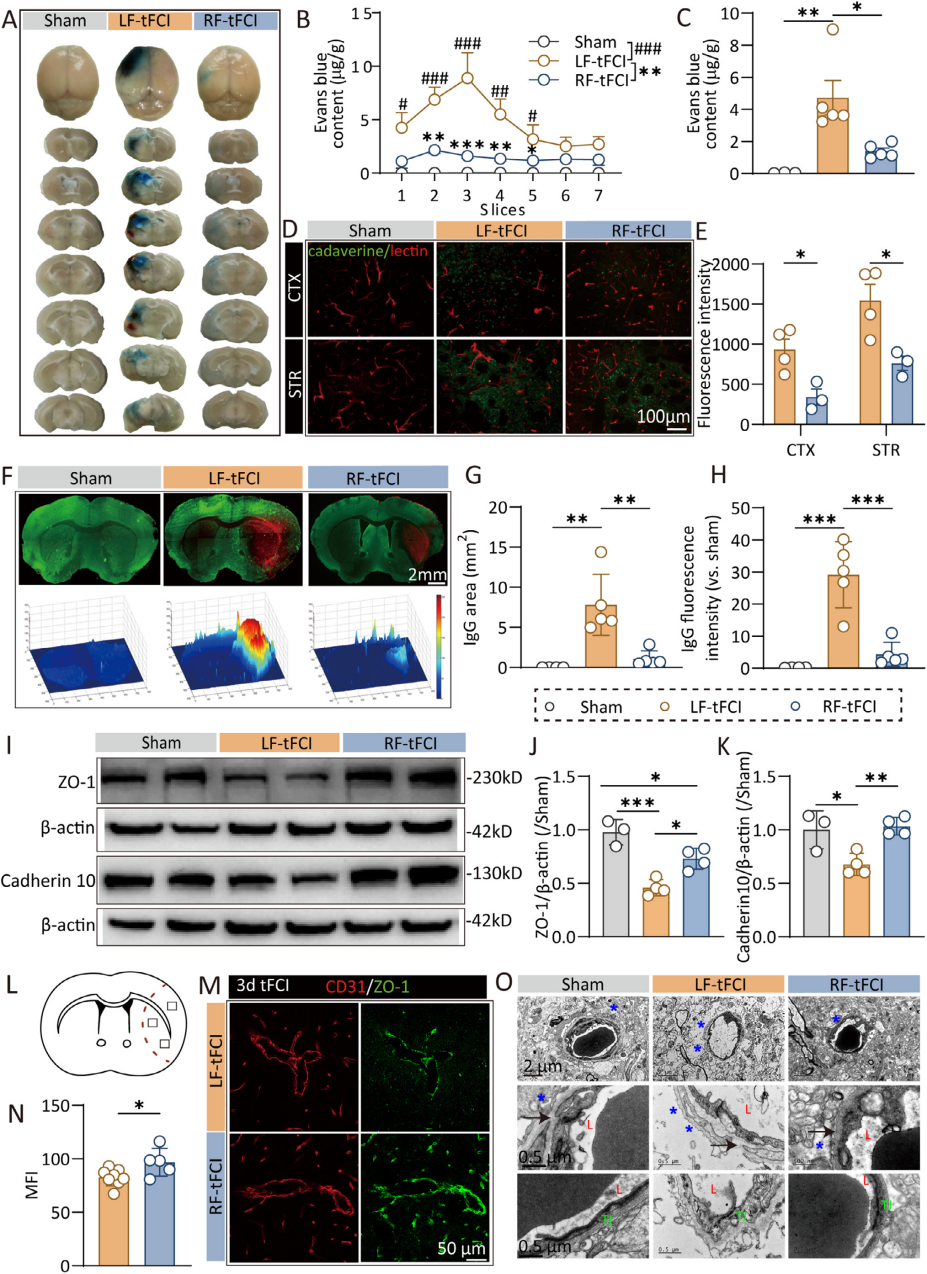
Caloric restriction protects the blood–brain barrier integrity after ischemic stroke. (A) Images of EB extravasation 48 h after tFCI. (B, C) The content of EB dye in each layer from rostral to caudal (B) and in the whole brain (C). *n* = 5/group. (D) Representative images of double immunostaining of cadaverine and lectin in cortex and striatum 48 h after tFCI. (E) Quantification of fluorescence intensity of cadaverine. *n* = 3–4/group. (F) The immunofluorescence images and 3D reconstruction images of IgG 48 h after tFCI. (G, H) Quantification of the leaked area (G) and fluorescence intensity (H) of IgG. *n* = 5/group. (I–K) The western blot images (I) and the relative expression (J, K) of ZO‐1 and cadherin 3 days after tFCI. *n* = 3–4/group. (L) Schematic diagram indicating the regions where images were captured in (M). (M) Representative images of CD31/ZO‐1 immunofluorescence 3 days after tFCI. (N) Quantification of fluorescence intensity of ZO‐1. *n* = 5 (RF‐tFCI group) or 8 (LF‐tFCI group). (O) Electric microscopy images of blood–brain barrier 48 h after tFCI. L: Capillary lumen; TJ: tight junction; *Astrocyte end‐feet; arrow: basement membrane. All data are presented as means ± SEM. Data were analyzed using unpaired two‐tail Student's *t*‐test (E, N), one‐way (J, K) or two‐way (B) ANOVA followed by Bonferroni post hoc test, or Kruskal–Wallis test with Dunn's multiple‐comparison post hoc test (C, G, H). *p < 0.05, **p < 0.01, ***p < 0.001, ^#^ *p* < 0.05, ^##^ *p* < 0.01, and ^###^ *p* < 0.001, as indicated.

The BBB disruption is a key pathophysiological alteration after ischemic stroke. Flow cytometry analysis (Figure 3A–F) demonstrated that RF led to a reduction in immune cell infiltration into the brain following tFCI, indicating its efficacy in preserving the integrity of BBB. To assess BBB integrity, we administered EB dye, which binds to plasma albumin and has a molecular weight of 68.5 kDa, via the femoral vein 48 h postischemia/reperfusion. EB leakage, observed after a 3‐h circulation, served as a marker for BBB disruption. We observed significant BBB injury in LF mice following ischemia/reperfusion, whereas RF markedly reduced EB leakage (Figure 4A–C; Figure S5). Additionally, we injected cadaverine (690 Da), another fluorescent dye, into the femoral vein and performed a 3‐h circulation. Lectin was injected into the left ventricle before perfusion to label functional vessels. Cadaverine diffusion was evident in the peri‐infarct cortex and striatum poststroke; however, RF limited cadaverine diffusion, indicating enhanced BBB integrity in RF mice (Figure 4D,E). Three days post‐tFCI, we evaluated IgG (150 kDa) leakage and found a significant reduction in both the area and intensity of leakage in RF mice compared to LF mice (Figure 4F–H). Moreover, western blot analysis revealed that the expression levels of the brain endothelial tight junction protein ZO‐1 and the intercellular junction protein cadherin 10, markers of BBB integrity, were elevated in RF mice compared to LF mice in the infarcted area 3 days post‐tFCI (Figure 4I–K). Immunofluorescence staining confirmed that the ZO‐1 expression in the vascular endothelium was elevated in the RF group compared to the LF group (Figure 4L–N).

Electron microscopy revealed that in LF mice, astrocytes were swollen and ruptured, the basement membrane was compromised, and tight junctions were severely damaged. In contrast, in RF mice, the BBB structure remained relatively intact, with reduced swelling of astrocyte end feet and relatively intact basement membrane and tight junction structures (Figure 4O). These findings indicate that caloric restriction significantly preserves BBB integrity during the acute phase of ischemic stroke.

### 3.5 Ly6G Mimics the Neuroprotective Effects of RF in Ischemic Stroke

**FIGURE 5:**
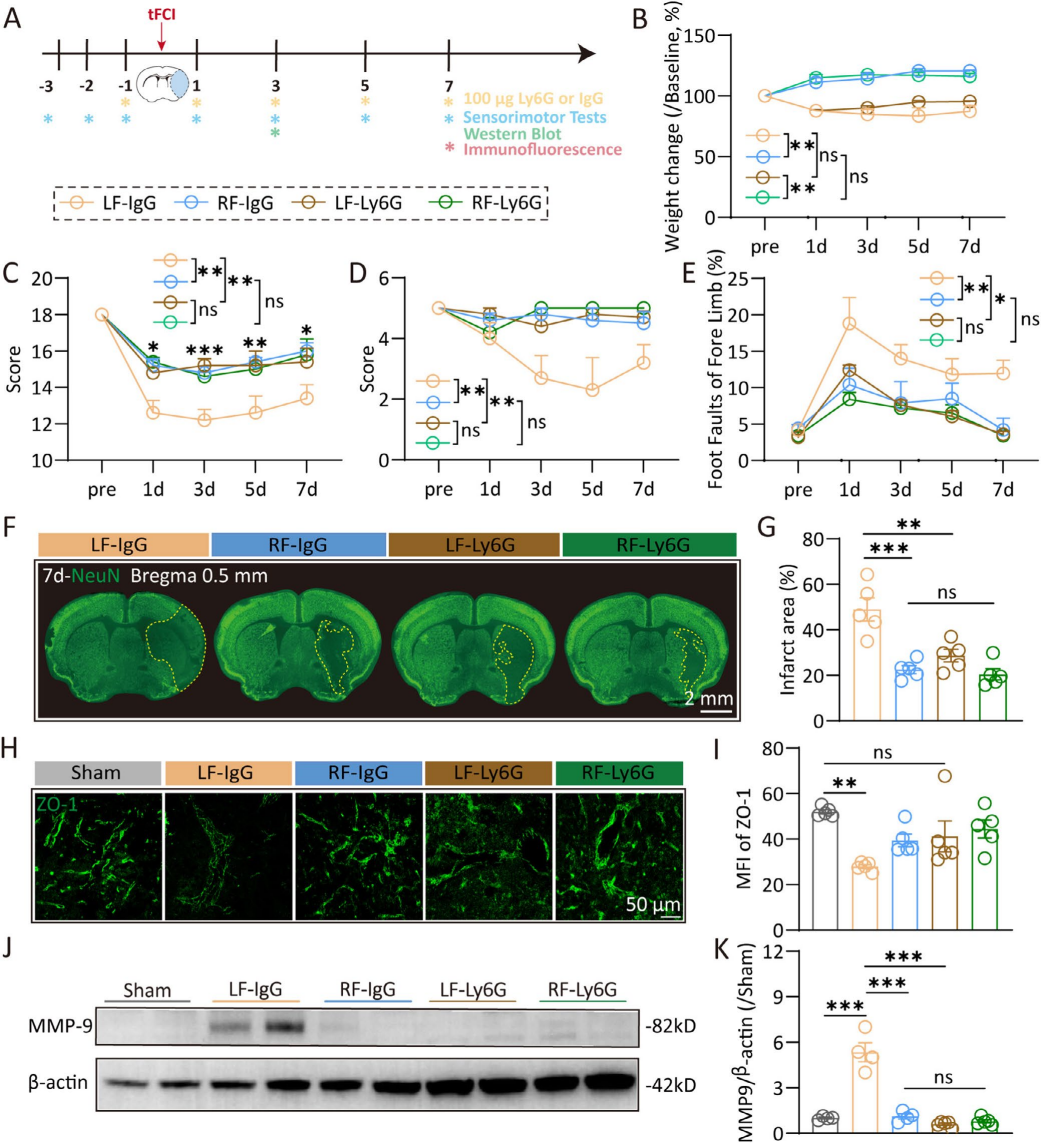
Reducing neutrophil infiltration may contribute to the protective effects of caloric restriction in ischemic stroke. (A) Diagram illustrating the spatiotemporal strategy for Ly6G injection and the following functional and histological measurements. (B) Weight change (vs. weight before surgery, %) after tFCI. Sensorimotor deficits were evaluated before (pre) and up to 7 days after tFCI by Garcia score (C), hang‐wire score (D), and foot faults test (E). *n* = 5/group. (F, G) Representative images (F) and the quantification of the infarct area (G) 7 days after tFCI. *n* = 5/group. (H, I) Representative images (H) and the mean immunofluorescent intensity (I) of ZO‐1 in the infarct core. (J‐K) Western blot of the penumbra and core tissue 3 days after tFCI were probed with anti‐MMP9 and β‐actin. *n* = 4–5/group. All data are presented as means ± SEM. Data were analyzed using one‐way (G, K) or two‐way (B–E) ANOVA followed by Bonferroni post hoc test, or Kruskal–Wallis test with Dunn's multiple‐comparison post hoc test (I). *p < 0.05, **p < 0.01, and ***p < 0.001, ns, no significance, as indicated.

Neutrophils are the initial immune cells to invade the brain after an ischemic stroke. Previous studies have demonstrated that neutrophils can impair BBB integrity and affect vascular remodeling during stroke recovery. Figure 3F,M showed that RF mice exhibited the less neutrophil infiltration in the brain compared to LF mice, prompting an investigation into whether this reduction is a key mechanism by which RF confers protection against ischemic stroke. To explore this, we treated mice with 100 μg of anti‐Ly6G antibody or an IgG isotype control intraperitoneally to block neutrophil infiltration, starting the day before tFCI and continuing every other day (as the experiment diagram illustrated in Figure 5A). The anti‐Ly6G antibody effectively blocked neutrophil infiltration as our previous study shows [30]. The treatment of anti‐Ly6G (RF‐Ly6G) did not change the mice's weight, while the RF mice kept a higher change in body weight than LF mice following tFCI (Figure 5B). Interestingly, LF mice treated with anti‐Ly6G (LF‐Ly6G) performed less dysfunctions compared to the LF‐IgG mice, resembling the neurological dysfunctions of RF mice, while the RF mice with anti‐Ly6G treatment (RF‐Ly6G) exhibited similar dysfunctions to the RF‐IgG mice (Figure 5C–E). The infarct area of LF‐Ly6G mice was reduced compared to LF‐IgG mice (Figure 5F,G), while RF‐Ly6G mice did not provide further less infarct than RF‐IgG mice. Furthermore, the tight junction protein ZO‐1 was significantly less in the LF‐IgG group than in the sham group, while there was no significant difference between the LF‐Ly6G group and both the sham and RF groups, suggesting that ZO‐1 degradation was effectively reduced in the LF‐Ly6G group (Figure 5H,I). Meanwhile, MMP‐9 expression level in LF‐Ly6G mice was much lower than that in LF‐IgG mice (Figure 5J,K; Figure S6). No statistically significant differences were observed in infarct volume, ZO‐1 expression, or MMP‐9 expression between the RF‐Ly6G and RF‐IgG mice. In summary, the findings indicate that inhibiting neutrophil infiltration into the brain after ischemic stroke can mimic the protective effects of RF on BBB integrity and neurological function. This further implies that the neuroprotective effects of RF are likely mediated through the inhibition of neutrophil infiltration into the brain and the subsequent release of MMP9.

## 4 Discussion

Caloric restriction preconditioning has been beneficial to various neurological disorders, including neurodegenerative and cerebrovascular diseases [13]. While earlier research has shown that RF safeguards white and gray matter after ischemic stroke [18], the exact mechanisms of these protective effects are not yet fully elucidated. Our study extends these findings by revealing that RF mitigates BBB damage after ischemic stroke. Furthermore, the protective effects of RF may be mediated through the reduction in chemokine secretion thus peripheral neutrophil infiltration, highlighting a novel mechanism by which RF confers neuroprotection in ischemic stroke.

During the acute phase following tFCI, breakdown of the BBB allows circulating immune cells and plasma‐derived molecules to infiltrate the ischemic brain parenchyma. This infiltration contributes to edema, inflammation, and secondary brain hemorrhage, thereby exacerbating brain damage [25, 34, 35]. RF has been shown to improve cerebral vascular health by reducing oxidative stress, enhancing nitric oxide bioactivity, and suppressing vascular inflammation. Key molecules and pathways involved in RF include maintaining vascular homeostasis, such as mTOR, AMPK, and endothelial nitric oxide synthase [20, 22, 23, 24]. In an aging mouse model, RF was observed to preserve CBF at 20 months of age compared to age‐matched mice on a normal diet. In another high‐fat diet mouse model, RF pretreatment was found to attenuate BBB leakage. Additionally, RF likely modulates short‐chain fatty acid levels in the intestinal microbiota, thereby enhancing tight junction protein expression in brain endothelial cells. These findings suggest that caloric restriction positively influences both the integrity and functionality of the BBB. Our study demonstrated that RF reduced leakage of molecules from circulation to the ischemic brain, including cadaverine, EB dye, and IgG, during the acute phase following tFCI. Additionally, RF preserved higher levels of tight junction protein ZO‐1 and intercellular junction protein cadherin 10, which was consistent with the observation of a more intact BBB as seen in electron microscopy. MMP9, an enzyme that promotes proteolysis of the BBB basal lamina and whose expression was significantly elevated after tFCI, was also mitigated by preconditioning. Furthermore, RF reduced the infiltration of circulating immune cells into the brain parenchyma after tFCI, indirectly suggesting enhanced BBB integrity. Collectively, our data indicate that RF effectively protects BBB integrity following ischemic stroke.

RF has been shown to reverse the accumulation of proinflammatory cells across multiple tissues in the body with aging [21]. Consequently, we wanted to investigate whether caloric restriction could modulate neuroinflammation following ischemic stroke. During ischemic stroke, microglia, as the resident immune cells of the brain parenchyma, are among the first responders to the injury. Microglia secrete both pro‐inflammatory cytokines, which exacerbate cell death and tissue injury, and anti‐inflammatory cytokines, which facilitate cell growth and regeneration [31, 36, 37, 38]. In line with our hypothesis, RF reduced the expression of the microglial proinflammatory marker CD16/32 as well as several proinflammatory cytokines induced by tFCI, including *Il‐6, Tnf‐α, Il‐16*, and *Nos2*. In addition to inflammatory cytokines, chemokines are small signaling proteins that contribute to the inflammatory processes associated with ischemic stroke. We observed an increase in the expression of chemokines such as *Ccl2, Ccl3, Ccl7, and Ccl22* following tFCI. However, RF significantly attenuated the upregulation of these chemokines. These findings suggest that RF effectively downregulates inflammation following ischemic stroke.

We observed substantial infiltration of immune cells, including neutrophils and macrophages, into the brain parenchyma following tFCI, as detected by flow cytometry. RF significantly reduced the infiltration of neutrophils, macrophages, and inflammatory macrophages, which aligns with our qPCR data showing reduced chemokine expression after ischemic stroke. Neutrophils are among the first immune cells to infiltrate the brain following ischemic stroke. The neutrophil extracellular traps formed due to neutrophil infiltration impede cerebrovascular remodeling and functional recovery during the later stages poststroke [39]. Conversely, neutrophil depletion has been shown to reduce BBB breakdown and enhance neovascularization [40, 41]. In an aging model, RF was found to mitigate the age‐related increase in neutrophils across multiple tissues [22]. Both fasting and RF promote the migration of immune cells to the bone marrow, thereby reducing circulating immune cells while preserving the capacity to respond to acute inflammation [42, 43]. These studies suggest that RF may modulate immune response, which is also indicated in our study. We observed that RF led to fewer neutrophils infiltrating the brain and reduced MMP9 protein expression after tFCI.

Furthermore, blocking neutrophil infiltration with anti‐Ly6G antibody resulted in improvements in sensorimotor function and BBB integrity in LF mice, consistent with our previous research and other studies [30, 40]. No significant difference in sensorimotor function or BBB integrity was observed between LF‐Ly6G and RF‐Ly6G groups, or between RF‐Ly6G and RF‐IgG groups. This indicated that the protective effects of RF were primarily attributable to its role in reducing neutrophil infiltration, rather than other mechanisms.

There are some limitations in our study. First, previous studies reported that RF promotes immune cells to migrate from circulation into bone marrow. However, we did not compare the immune cell composition in LF sham with that in RF sham. This is because what we focus on in this study is the preventative effects of RF against ischemic stroke. Therefore, mice in RF group also had full access to food after tFCI. On the third day to perform flow cytometry, there is no difference between LF sham and RF sham. Second, although we observed that RF secreted fewer chemokines and recruited fewer neutrophils, which may be the mechanism of RF's protective effects on tFCI, we did not elucidate the mechanism underlying this phenomenon. Third, numerous preclinical studies have failed to demonstrate clinical translation because they used only male animals. Gender dimorphisms have been reported in the brain structure, metabolism, function, and response to stroke. The specific effects of RF following tFCI in female animals should be examined in subsequent research. Finally, flow cytometry was employed to identify inflammatory cell infiltration in the brain 3 days post‐tFCI. The results indicated a significant increase in macrophage infiltration, while RF notably inhibited this infiltration. Consequently, further investigation is warranted to determine whether RF similarly mediates neuroprotective effects and facilitates neurological recovery through the modulation of macrophages.

In conclusion, our study confirmed that caloric restriction effectively mitigates brain damage postischemic stroke and uncovered its mechanism in preserving BBB integrity by reducing neutrophil infiltration, thereby broadening the understanding of RF's protective effects on ischemic stroke.

## Author Contributions

Y.G. designed this study. C.W., L.M., M.H., J.Z., Y.H., and Y.Z. performed experiments. C.W., L.M., and J.X. analyzed the data. C.W. and L.M. wrote the manuscript. Y.G. and S.H. critically edited the manuscript. The authors read and approved the final manuscript.

## Conflicts of Interest

The authors declare no conflicts of interest.

## Supporting information


Figures S1–S6.

## Data Availability

The data that support the findings of this study are available from the corresponding author upon reasonable request.
