# Supplementary material for: Caloric Restriction Preserves BBB Integrity After Transient Focal Cerebral Ischemia Through Reducing Neutrophil Infiltration
Source: CNS Neurosci Ther. 2025 Feb 6;31(2):e70257. doi: 10.1111/cns.70257 (PMC11802461; doi:10.1111/cns.70257)
Supplement: Supplementary file 1 — Figures S1–S6. [file CNS-31-e70257-s001.pdf]

Caloric restriction preserves BBB integrity after transient focal cerebral ischemia through reducing neutrophil infiltration

Chenran Wang[$], Leilei Mao[$], Miao He, Jia Zhang, Yichen Huang, Yue Zhang, Jing Xu*, Shaoqiang Huang*, Yanqin Gao*

Department of Anesthesiology of Eye & Ent Hospital, Department of Anesthesiology of Obstetrics & Gynecology Hospital, State Key Laboratory of Medical Neurobiology, MOE Frontiers Center for Brain Science, and Institutes of Brain Science, Fudan University, Shanghai, China.

[$]These authors contributed equally to this work.

*Corresponding author. Yanqin Gao (yqgao@shmu.edu.cn), Jing Xu (shxujing1234@hotmail.com), and Shaoqing Huang (drhuangsq@fudan.edu.cn)

Address: 138 Yi Xue Yang Road, Xuhui District, Shanghai, China, 200032.

Supplementary Figures

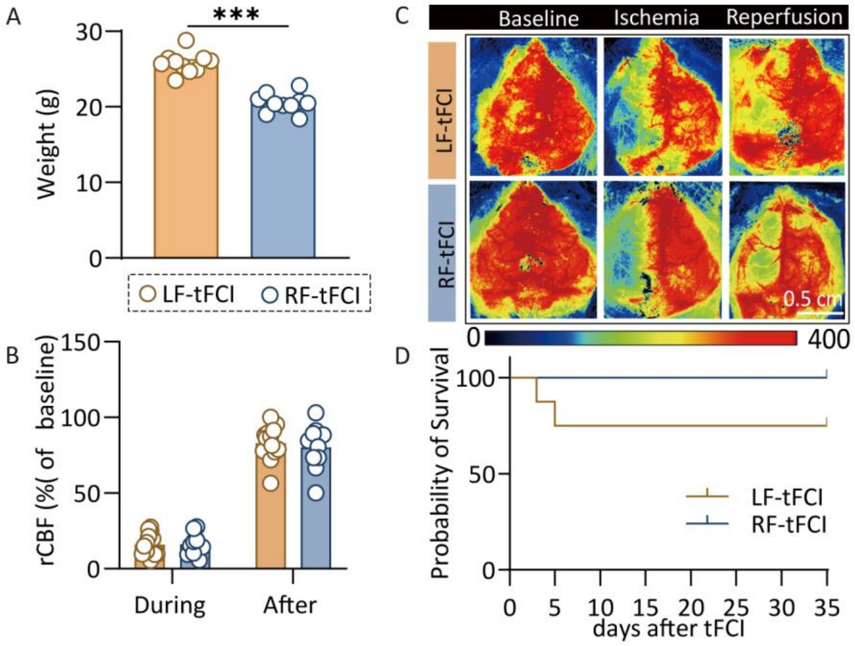

Figure S1. Caloric restriction reduces the body weight but not change the relative cerebral blood flow in tFCI. (A) The body weight of mice before tFCI. n=10/group. (B) Relative cerebral blood flow during and after tFCI. n= 10 (RF-tFCI group) or 14 (LF-tFCI group). (C) Representative images of the cerebral blood flow before, during and after tFCI. (D) The survival curve of tFCI model. n= 6 (RF-tFCI group) or 8 (LF-tFCI group). All data are presented as means±SEM. Data were analyzed using unpaired two-tails Student's t-test, ***p<0.001, as indicated.

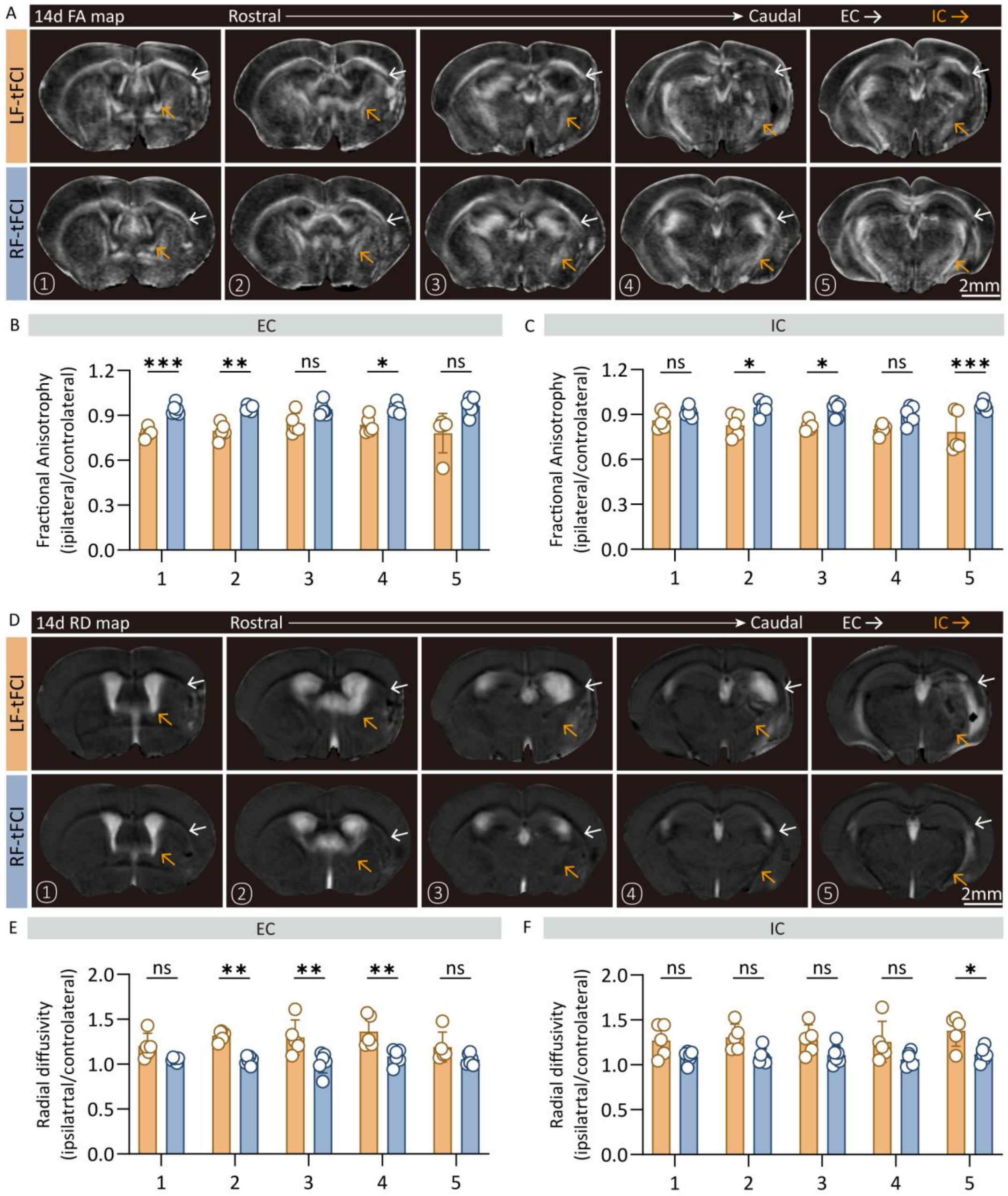

Figure S2. *ex vivo* DTI-MRI on day 14 after tFCI, related to Figure 1. (A) FA map of *ex vivo* DTI-MRI presented from rostral planes to caudal planes（B-C) Quantification of FA value on different planes in the EC (B) or IC (C). n=5/group. (D) RD map of *ex vivo* DTI-MRI presented from rostral planes to caudal planes. (E-F) Quantification of RD value on different planes in the EC (E) or IC (F). n=5/group. All data are presented as means±SEM. Data were analyzed using two-way ANOVA with Bonferroni post hoc tes, *p<0.05, **p<0.01, ***p<0.001, as indicated.

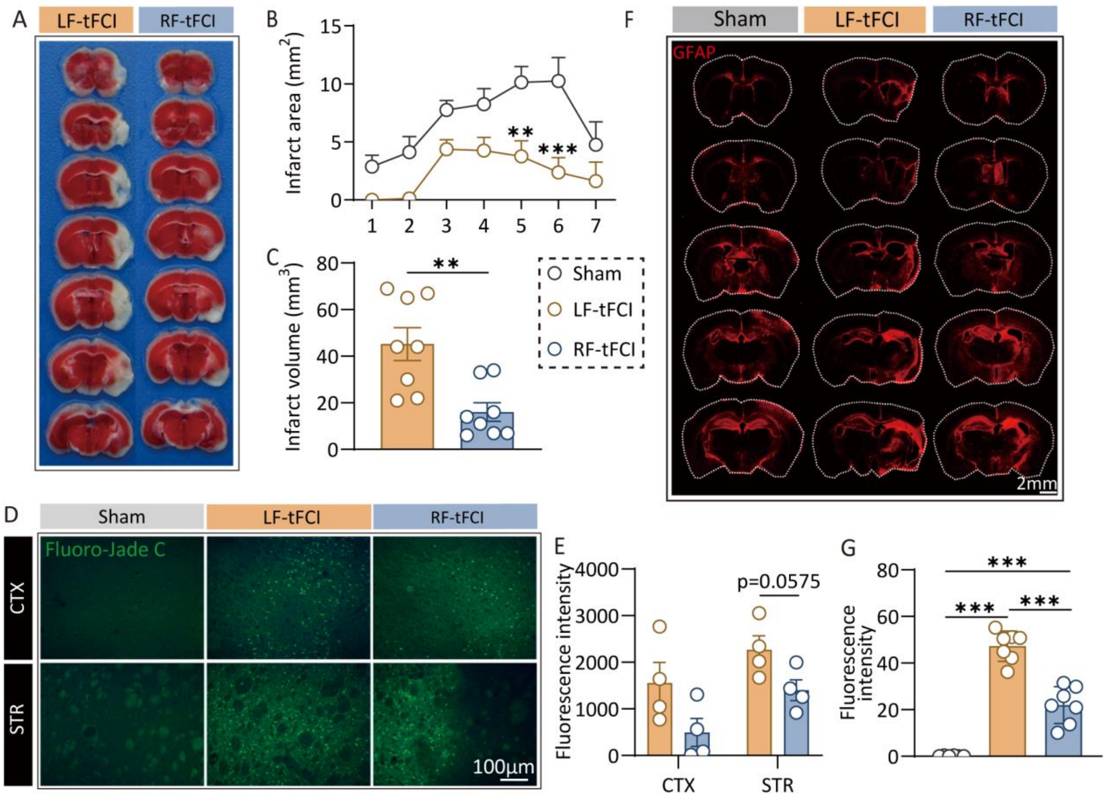

Figure S3. Caloric restriction ameliorates histological deficits after ischemic stroke, related to Figure 1. (A) Triphenyl tetrazolium chloride (TTC) staining at 48h after 60-min tFCI. (B) The infarct area of TTC staining of each brain slice from rostral to caudal. (C) The total infarct volume of TTC staining. n=8/group. The representative images (D) and the fluorescence intensity (E) of Fluoro-Jade C staining at 48h after tFCI. n=4/group. The images (F) and the fluorescence intensity (G) of GFAP staining 28d after tFCI. n=6-7/group. All data are presented as means±SEM. Data were analyzed using unpaired two-tails Student's t-test (B, E), or Mann Whitney test (C), or one-way ANOVA followed by Bonferroni post hoc test (G). ***p*<0.01, ****p*<0.001, as indicated.

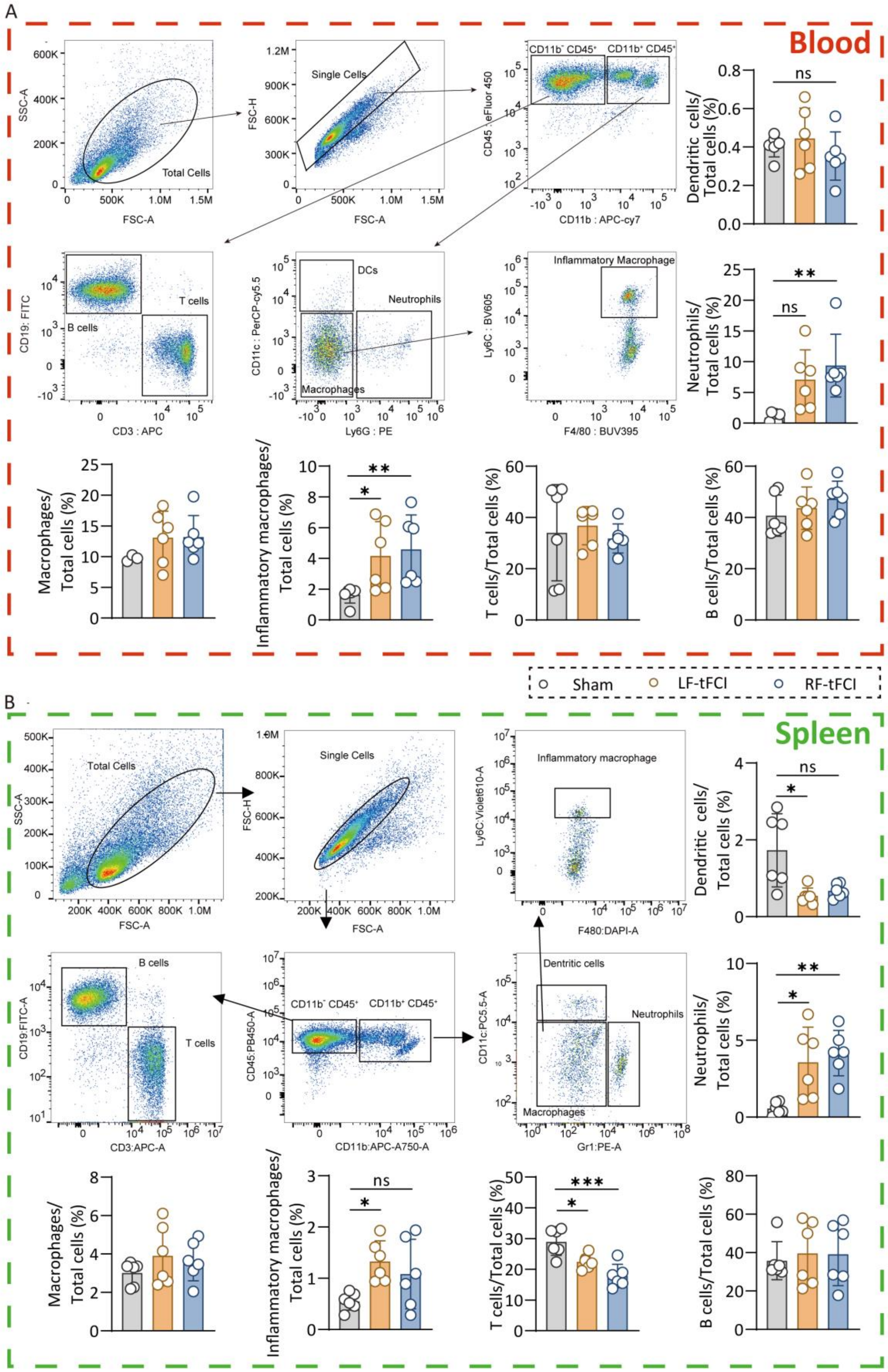

Figure S4. Caloric restriction mildly affects peripheral immunity after ischemic stroke. (A) The gating strategy for flow cytometry of blood cells and the quantification

results of several immune cell types in blood. (B) The gating strategy for flow cytometry of spleen cells and the quantification results of several immune cell types in spleen. n=6/group. All data are presented as means ± SEM. Data were analyzed using one-way ANOVA followed or Kruskal-Wallis test by Bonferroni post hoc test. *p<0.05, **p<0.01, ***p<0.001, ns: no significance, as indicated.

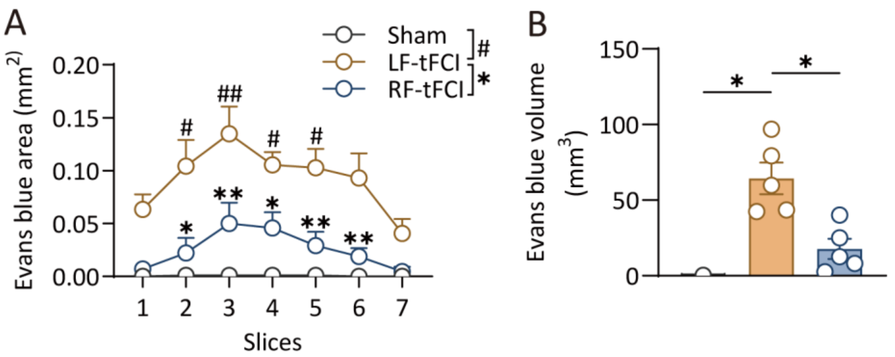

Figure S5. Caloric restriction markedly reduced Evans blue leakage following ischemia/reperfusion, related to Figure 4. The area of Evans blue dye in each layer from rostral to caudal (A) and the total volume of Evans blue dye (B). n=5/group. All data are presented as means±SEM. Data were analyzed using one-way ANOVA followed by Bonferroni post hoc test. *p< 0.05, **p< 0.01, $^{\#}p$< 0.05, $^{\#\#}p$< 0.01, as indicated.

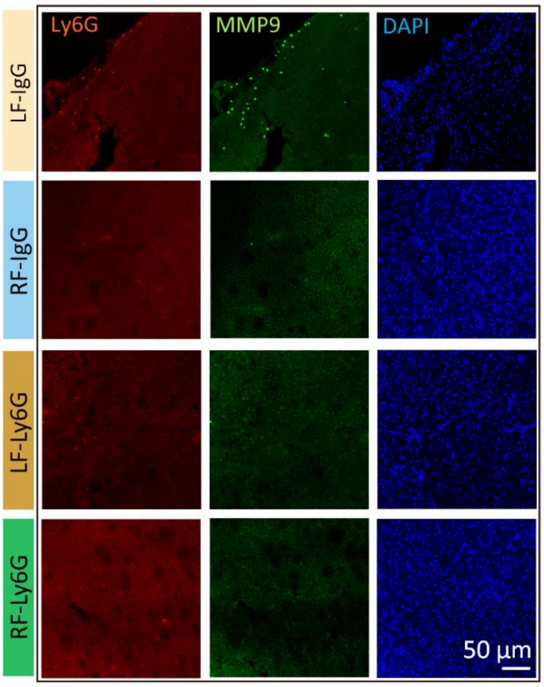

Figure S6. Ly6G mimics the MMP-9 inhibition effect of RF on ischemic stroke, related to Figure 5. Representative images of double immunostaining of Ly6G and MMP9 at 7 days after tFCI.
